# Bubbles Don’t Lie: Superior Vena Cava Thrombosis With a Systemic-to-Pulmonary Venous Shunt Mimicking a Patent Foramen Ovale

**DOI:** 10.7759/cureus.117756

**Published:** 2026-10-11

**Authors:** Aurelien Guitton, Dulcis Burundi, Mihai Strachinaru

**Affiliations:** 1 Cardiology, Vivalia Arlon Hospital, Arlon, BEL; 2 Radiology, Erasmus Hospital, Brussels, BEL; 3 Cardiology, Erasmus Hospital, Brussels, BEL

**Keywords:** extracardiac right-to-left shunt, paradoxical embolism, patent foramen ovale, superior vena cava thrombosis, transesophageal echocardiography

## Abstract

In patients with ischaemic stroke, right-to-left shunting on a bubble study is often attributed to a patent foramen ovale (PFO) and taken as sufficient grounds for percutaneous closure. We report the case of a 60-year-old woman admitted for an ischaemic stroke, in whom transoesophageal echocardiography (TOE) showed microbubbles in the left atrium after right antecubital injection of agitated saline. A PFO was presumed, and the patient was referred for transcatheter closure.

During the procedure, repeated attempts to cross the interatrial septum failed. Repeat TOE with agitated saline injected directly adjacent to the interatrial septum showed no septal transit. A subsequent left antecubital injection produced left atrial opacification as abundant as the right-sided injection, with microbubbles consistently entering through the pulmonary veins. This excluded an interatrial shunt and pointed toward a large, fixed, extracardiac right-to-left conduit between the systemic venous return and the pulmonary venous circulation. The closure procedure was therefore abandoned.

Contrast-enhanced computed tomography (CT) angiography resolved the discrepancy between the positive bubble study and the absence of an interatrial communication. It showed a superior vena cava thrombosed around a long-standing central venous catheter, with extensive venous collateralisation: the left brachiocephalic vein drained through a network of collaterals into a markedly dilated azygos vein. Large-calibre collaterals anastomosed directly with the pulmonary veins, creating a haemodynamically significant right-to-left pathway into the left atrium that bypassed the pulmonary capillary filter and provided the substrate for paradoxical embolism. After multidisciplinary discussion, the patient was treated with long-term oral anticoagulation with a vitamin K antagonist.

This case highlights that a positive bubble study is not synonymous with a PFO. Identifying the site of microbubble entry into the left atrium, and performing bilateral arm injections when findings are atypical or septal crossing fails, can help distinguish an intracardiac from an extracardiac shunt and prevent unnecessary intervention. In patients with a history of central venous catheterisation, superior vena cava thrombosis with collaterals draining into the pulmonary veins is a rare cause of right-to-left shunting and paradoxical embolism, and venous imaging may be considered before closure.

## Introduction

Paradoxical embolism through a right-to-left shunt is an established mechanism of ischaemic stroke, particularly in patients in whom no other embolic source is identified. Under normal conditions, the pulmonary capillary bed acts as a filter: small venous thrombi are trapped in the pulmonary microcirculation and lysed there, and do not reach the systemic arterial circulation. A right-to-left shunt bypasses this filter. Patent foramen ovale (PFO) is the most common substrate, and contrast echocardiography with agitated saline remains the cornerstone of its detection, often guiding the decision for percutaneous closure [1,2]. Agitated saline contains air microbubbles that are strongly echogenic but do not cross the normal pulmonary capillary bed. After peripheral venous injection, they opacify the right heart chambers only, so their appearance in the left heart indicates a right-to-left shunt [3].

However, left-heart opacification demonstrates the presence of a shunt, not its location. Three mechanisms must be distinguished. In intracardiac shunts (PFO, atrial septal defect), microbubbles cross the interatrial septum early after right atrial opacification. In intrapulmonary shunts (pulmonary arteriovenous malformation, hepatopulmonary syndrome), they enter through the pulmonary veins after a delay of several cardiac cycles. In the much rarer extracardiac systemic-to-pulmonary venous communications, they enter through the pulmonary veins after bypassing both the right heart and the pulmonary capillary bed, with a timing that depends on the collateral route [4-6]. The last two may be mistaken for an interatrial shunt.

Acquired systemic-to-pulmonary venous collaterals are an uncommon consequence of chronic superior vena cava obstruction, most often related to central venous catheters or intravascular devices. When venous return is diverted through mediastinal collaterals that drain into the pulmonary veins, deoxygenated blood reaches the left atrium while bypassing the pulmonary capillary filter, creating a route for paradoxical embolism [4,5].

We report the case of a patient with ischaemic stroke referred for PFO closure after a positive bubble study, in whom a careful reassessment during the procedure revealed an extracardiac right-to-left shunt caused by catheter-related superior vena cava thrombosis. The aim of this report is to show that a positive bubble study does not localise the shunt, and to illustrate how the site of microbubble entry on transoesophageal echocardiography, bilateral arm injections, and computed tomography angiography distinguish an extracardiac venous shunt from a presumed PFO.

## Case presentation

A 60-year-old woman with a history of hypertension, dyslipidaemia, chronic kidney disease, and triple-negative breast cancer treated with chemotherapy was admitted for an acute ischaemic stroke. She presented with aphasia, and the National Institutes of Health Stroke Scale (NIHSS) score on admission was 3. Brain magnetic resonance imaging showed an acute infarct in the left middle cerebral artery territory. On admission, blood pressure was 135/81 mmHg, heart rate 86 bpm, and oxygen saturation 97% on room air. There were no clinical signs of superior vena cava syndrome or of right-to-left shunting.

A central venous catheter had been inserted for chemotherapy at least five years earlier and was still in place.

The aetiological work-up included carotid duplex ultrasound, which was normal, and cardiac rhythm monitoring, which showed no atrial fibrillation; it did not identify an alternative embolic source. Routine coagulation tests were normal apart from elevated D-dimer levels. Thrombophilia screening and arterial blood gas analysis were not performed.

Transoesophageal echocardiography (TOE) was performed under conscious sedation with 2 mg of midazolam and local anaesthesia with lidocaine. Agitated saline was injected through a right antecubital vein, with imaging at a multiplane angle of 80-90° at rest; a Valsalva manoeuvre was not performed. Microbubbles appeared in the left atrium about 12 cardiac cycles after injection (Video 1). This was interpreted as a right-to-left shunt through a PFO, and the patient was referred for percutaneous closure.

**Video 1:** Initial transoesophageal echocardiography with right arm injection Microbubbles transit into the left atrium following right antecubital agitated saline injection.

During the closure procedure, repeated attempts to cross the interatrial septum with the catheter failed. Repeat TOE was performed with agitated saline injected directly adjacent to the interatrial septum, which showed no septal transit of microbubbles despite adequate right atrial opacification (Video 2).

**Video 2:** Transoesophageal echocardiography with injection adjacent to the interatrial septum No septal microbubble transit despite adequate right atrial opacification.

A subsequent left antecubital injection, imaged in the same view, produced left atrial opacification as abundant as the right-sided injection. Microbubbles consistently entered through the left superior pulmonary vein rather than across the septum, again about 12 cardiac cycles after injection (Video 3). These findings excluded an interatrial shunt and pointed toward a large, fixed, extracardiac right-to-left communication between the systemic venous return and the pulmonary venous circulation (Table 1). The closure procedure was therefore abandoned.

**Video 3:** Transoesophageal echocardiography with left arm injection Abundant microbubble transit into the left atrium, entering through the left superior pulmonary vein.

**Table 1:** Differential diagnosis of left-heart opacification after agitated saline injection PFO: patent foramen ovale

Mechanism	Main causes	Site of microbubble entry into the left atrium	Timing	Other clues
Intracardiac shunt	PFO, atrial septal defect	Across the interatrial septum	Early: within three to six cardiac cycles of right atrial opacification	Increases with Valsalva manoeuvre (PFO); same result from either arm
Intrapulmonary shunt	Pulmonary arteriovenous malformation, hepatopulmonary syndrome	Through the pulmonary veins	Delayed: after more than three to six cardiac cycles	Not Valsalva-dependent; same result from either arm
Systemic-to-pulmonary venous shunt	Superior vena cava obstruction with collaterals to the pulmonary veins; persistent left superior vena cava draining into the left atrium	Through the pulmonary veins, or directly into the left atrium	Variable: depends on the collateral route; may precede right-heart opacification	No septal crossing; may depend on the injection site; history of central venous catheter or intravascular device

Contrast-enhanced computed tomography (CT) angiography was then performed, with iodinated contrast injected through a right peripheral venous line, acquisition in both the early first-pass and venous phases, and multiplanar reconstructions. The left brachiocephalic vein drained through an extensive network of mediastinal venous collaterals into a markedly dilated azygos vein (Figure 1).

**Figure 1:**
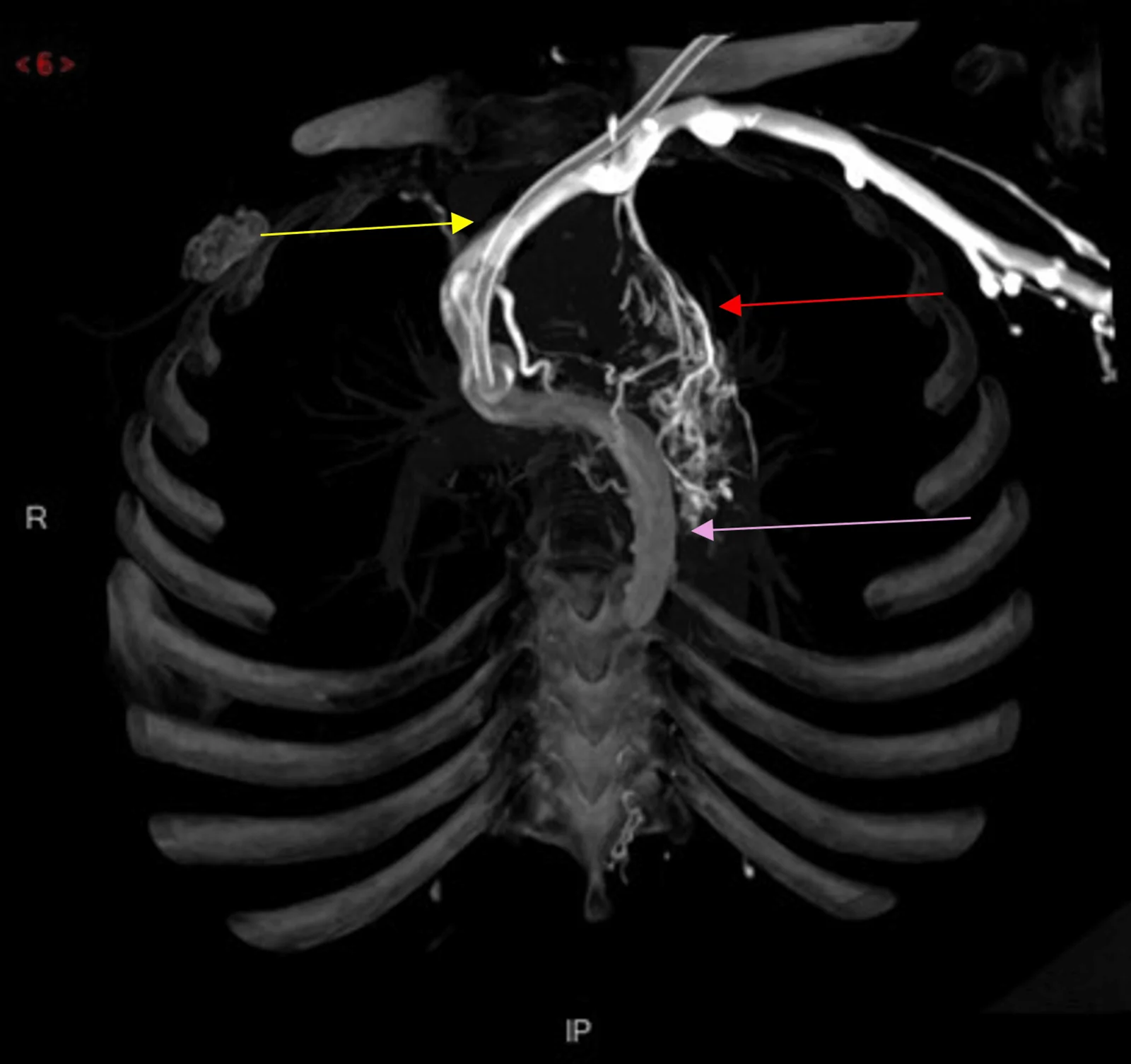
Coronal CT angiography reconstruction Left brachiocephalic vein (yellow arrow), venous collaterals (red arrow) and dilated azygos vein (pink arrow).

The superior vena cava was thrombosed along the entire length of the central venous catheter, and several large-calibre collaterals anastomosed directly with the pulmonary veins (Figure 2). This created a haemodynamically significant right-to-left pathway into the left atrium that bypassed the pulmonary capillary filter and was the most likely route of paradoxical embolism (Figure 3). No pulmonary arteriovenous malformation and no proximal pulmonary embolism were seen; ventilation-perfusion scintigraphy was not performed.

**Figure 2:**
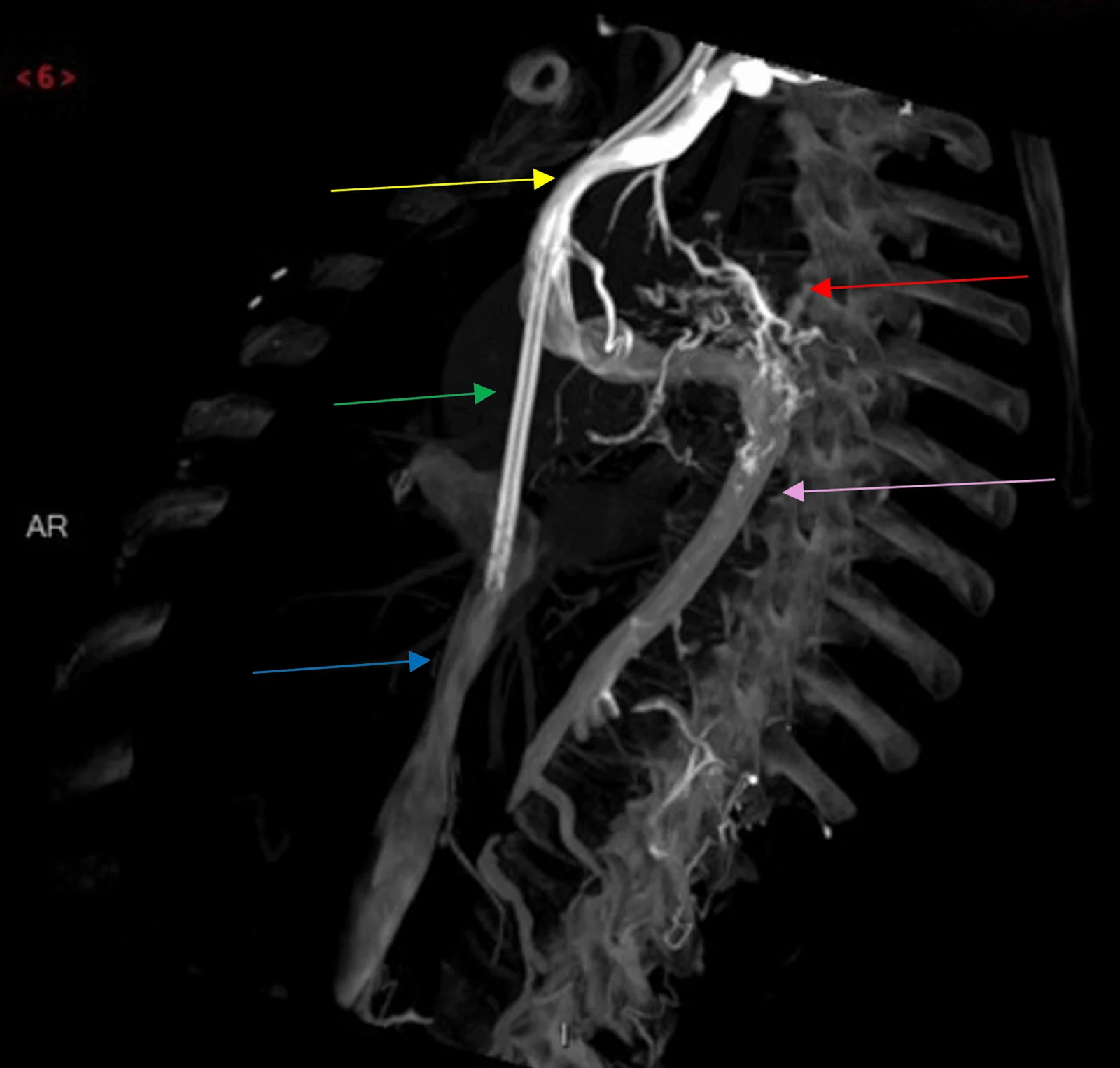
Oblique sagittal CT angiography reconstruction Left brachiocephalic vein (yellow arrow), venous collaterals (red arrow), thrombosed superior vena cava (green arrow), dilated azygos vein (pink arrow) and inferior vena cava (blue arrow).

**Figure 3:**
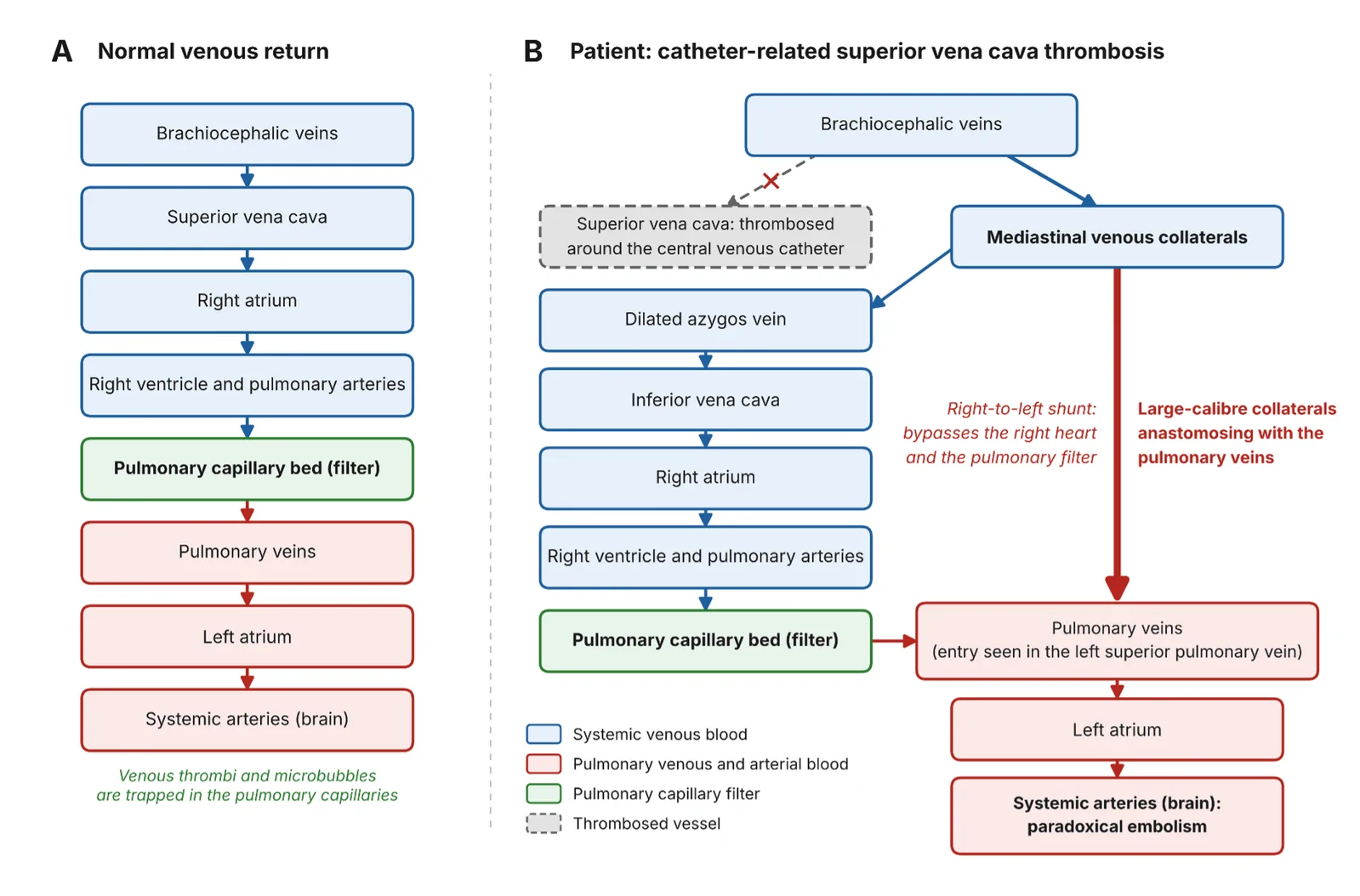
Schematic diagram of (A) normal venous return, and (B) the abnormal venous pathway in the patient In the patient, the superior vena cava is thrombosed around the central venous catheter. Venous return from the brachiocephalic veins is diverted through mediastinal collaterals, partly to the dilated azygos vein and partly, through large-calibre collaterals, directly to the pulmonary veins. This second route reaches the left atrium without passing through the pulmonary capillary filter.

The therapeutic strategy was discussed at a multidisciplinary team meeting. Endovascular recanalisation of the superior vena cava was not considered feasible because the thrombosis was too extensive, and the catheter was left in place. The patient was started on long-term oral anticoagulation with a vitamin K antagonist (target international normalised ratio 2.0-3.0) to address both the superior vena cava thrombosis and the risk of recurrent paradoxical embolism. A vitamin K antagonist was preferred to a direct oral anticoagulant because the latter has not been studied in this setting. No percutaneous or surgical intervention on the venous collaterals was undertaken. At 10 months of follow-up, the patient had had no recurrent stroke. No follow-up imaging has been performed, as she has since moved abroad.

## Discussion

This case illustrates a diagnostic pitfall in the evaluation of stroke patients with a positive bubble study. The appearance of microbubbles in the left atrium confirms the presence of a right-to-left shunt but does not, by itself, localise it. Current recommendations emphasise that the diagnosis of PFO relies on the demonstration of microbubble passage across the interatrial septum, ideally visualised directly on TOE [1,2,6]. In our patient, the initial study was interpreted as consistent with a PFO, which led to referral for closure.

Two simple manoeuvres made it possible to establish the correct diagnosis. First, a saline injection performed adjacent to the interatrial septum during the procedure showed no transseptal crossing, which was consistent with the repeated failure of catheter crossing. Second, a contralateral arm injection produced equally abundant left atrial opacification, with bubbles clearly entering through the pulmonary veins. An interatrial shunt would not be expected to show a pulmonary venous entry pattern, whereas an intrapulmonary shunt would typically produce delayed opacification, after several cardiac cycles [6] (Table 1). In our patient, microbubbles reached the left atrium about 12 cardiac cycles after peripheral injection. Because this delay was measured from injection and not from right atrial opacification, and because transit through a collateral network may itself be slow, timing alone could not distinguish an extracardiac from an intrapulmonary shunt. The diagnosis rested on the abundant pulmonary venous entry from either arm, the absence of septal crossing and the venous anatomy on CT. Bilateral arm injections, and careful identification of the site of bubble entry into the left atrium, may be considered whenever the findings are atypical or when septal crossing proves unexpectedly difficult. These manoeuvres are not proposed as a systematic step before every PFO closure, but as problem-solving tools when the echocardiographic findings and the expected anatomy are discordant.

Systemic-to-pulmonary venous collaterals are a rare but recognised consequence of chronic superior vena cava obstruction. When the superior vena cava is occluded, venous return from the upper body is diverted through mediastinal, azygos and chest wall collaterals. Some of these channels may communicate with the pulmonary veins, allowing systemic venous blood to reach the left atrium directly [4,5,7]. Central venous catheters are a recognised cause of such obstruction, through catheter-related thrombosis and progressive fibrotic occlusion of the vessel [4,8]. Similar cases of paradoxical stroke related to catheter-associated or brachiocephalic venous thrombosis with collateral drainage to the left atrium have been reported, but they remain exceptional [4,5,9]. In our patient, the long-standing catheter was the most likely trigger of superior vena cava thrombosis, and the resulting collateral network provided a direct route for paradoxical embolism.

Contrast-enhanced CT angiography was essential to characterise the anatomy of the shunt and to exclude alternative diagnoses such as a pulmonary arteriovenous malformation, the main cause of intrapulmonary right-to-left shunting [10], or an anomalous venous connection. Dynamic contrast-enhanced CT can directly depict the systemic-to-pulmonary venous pathway in superior vena cava obstruction [7]. Cross-sectional imaging is not part of the routine work-up before PFO closure, and in our patient it was obtained only after the failed procedure. In retrospect, the long-standing central venous catheter was a clue that could have prompted earlier venous imaging. In patients with a history of central venous catheterisation or other intravascular devices, cross-sectional venous imaging should be considered before PFO closure when the site or timing of microbubble entry is atypical.

Two levels of evidence should be distinguished in this report. The absence of septal crossing, the pulmonary venous entry of microbubbles and the venous anatomy on CT were directly observed. That the stroke resulted from paradoxical embolism through this pathway is an inference, supported by the absence of an alternative embolic source and by the coexistence of venous thrombosis and a large-calibre shunt. This report also has limitations: the timing of microbubble appearance was measured from peripheral injection and not from right atrial opacification, a Valsalva manoeuvre and thrombophilia screening were not performed, three-dimensional CT reconstructions were not available, and no follow-up imaging is available after 10 months of clinical follow-up.

Management options include long-term anticoagulation, removal of the catheter, endovascular recanalisation and stenting of the superior vena cava, or embolisation of the collateral vessels [4,5,9,11]. Intravascular devices now account for a substantial proportion of superior vena cava obstruction, and endovascular treatment is increasingly used in this setting [8,11]. Recanalisation may reduce venous hypertension and flow through the collaterals, but it is technically demanding in chronic occlusions. Embolisation is limited when the collateral network is diffuse. In our patient, the therapeutic strategy was discussed at a multidisciplinary team meeting, and long-term anticoagulation with a vitamin K antagonist was chosen to treat the venous thrombosis and reduce the risk of recurrent paradoxical embolism. Endovascular recanalisation was not retained because the thrombosis was too extensive, and a vitamin K antagonist was preferred to a direct oral anticoagulant because the latter have not been studied in this setting. Importantly, the benefit of PFO closure has been demonstrated only in patients in whom the PFO is the likely mechanism of stroke [12,13]. In our patient, closure would have been of no benefit and would have exposed the patient to unnecessary procedural risk.

## Conclusions

A positive bubble study demonstrates a right-to-left shunt but does not localise it. The central diagnostic step is to identify where microbubbles enter the left atrium: across the interatrial septum or through the pulmonary veins. When this entry is atypical or septal crossing fails, bilateral arm injections and CT angiography can reveal an extracardiac shunt. In patients with a history of central venous catheterisation, superior vena cava thrombosis with systemic-to-pulmonary venous collaterals is a rare cause of paradoxical embolism, and recognising it avoids unnecessary PFO closure.
